# Vinpocetine’s immunomodulating, anti-oxidant, anti-inflammatory, ant-ifibrotic, and PDE inhibiting potencies ameliorate bleomycin-induced pulmonary fibrosis

**DOI:** 10.22038/IJBMS.2022.64175.14130

**Published:** 2023-01

**Authors:** Mohamed Balaha, Abdullah Alahmari, Samah Kandeel, Marwa Balaha

**Affiliations:** 1 Clinical Pharmacy Department, College of Pharmacy, Prince Sattam bin Abdulaziz University, Al-Kharj, Saudi Arabia; 2 Pharmacology Department, Faculty of Medicine, Tanta University, El-Gish Street, Postal No. 31527, Tanta, Egypt; 3 Histology Department, Faculty of Medicine, Tanta University, El-Gish Street, Postal No. 31527 Tanta, Egypt; 4 Department of Pharmacy, University G. d’Annunzio, Chieti-Pescara, Italy; 5 Department of Pharmaceutical Chemistry, Faculty of Pharmacy, Kafrelsheikh University, Postal No. 33516, Kafr El Sheikh, Egypt

**Keywords:** Antifibrotic, Anti-inﬂammatory, Anti-oxidant, Pulmonaryfibrosis, Phosphodiesterase, Vinpocetine

## Abstract

**Objective(s):**

Pulmonary fibrosis (PF) is a global health problem with a high economic burden. Intratracheal administration of bleomycin is the best model that resembles the pathogenesis of PF in humans. Recently, vinpocetine proved to have neuroprotective, cardioprotective, hepatoprotective, anti-aging, and antifibrotic effects through its anti-oxidant, immunomodulating, and anti-inﬂammatory activities. The present study investigated the antifibrotic potentiality of vinpocetine in a rat model of PF induced by intratracheal bleomycin administration.

**Materials and Methods:**

PF induced by a single intratracheal instillation of 5 mg/kg bleomycin in nine-week-old Wister rats. Oral vinpocetine was used at doses of 5, 10, or 20 mg/kg to treat PF for 21 days immediately after the bleomycin instillation.

**Results:**

Vinpocetine dose-dependently ameliorates PF induced by bleomycin administration since vinpocetine effectively restored the normal body weight gain rates, pulmonary architecture, and collagen fiber distribution and suppressed the elevated BALF cell count, lymphocytes and neutrophils percentage, BALF, IL-6, TNF-α, and TGF-β1 levels and LDH activity, lung tissue MDA level, PDE activity, hydroxyproline content, immunohistochemical expression of α-SMA and CD68 positive macrophage, and fibrosis score. Meanwhile, it efficiently augmented the reduced BALF macrophage percentage, IL-10 level, lung tissue GSH level, CAT, and SOD activities.

**Conclusion:**

Vinpocetine may propose a new promising agent to manage PF.

## Introduction

Pulmonary fibrosis (PF) is a global health problem, affecting nearly 8-60 per 100,000 cases per year worldwide, with an incidence increasing to 200 per 100,000 cases per year after the age of 70 and increasing annually, with a high economic burden (1). PF is the ultimate consequence of many acute and chronic lung diseases in humans that cause damage to the lung parenchyma, which is characterized by an irreversible, chronic, and progressive disease course with permanent fibrosis, which ends fatally, with a median survival time of 3-5 years and 2-3 months, after diagnosis, or after an acute exacerbation, respectively (2). Clinically, PF is manifested by dry cough, progressive dyspnea, restrictive pulmonary dysfunction, hypoxemia, hypercapnia, heart, and respiratory failure, reflecting the underlying histopathological changes in lung tissues in the form of heterogeneous interstitial fibrosis, fibrotic foci, and parenchymal deformation (3). 

The exact etiology of PF remains unclear, but complex interplays between genetic susceptibility and environmental factors, such as aging, cigarette smoking, gastroesophageal reflux, diabetes mellitus, viral infection, and obstructive sleep apnea, are considered the main risk factors involved in the development of PF (2, 4). Furthermore, the underlying pathogenesis of PF is not yet fully understood; however, many mechanisms have been suggested, including prolongation of the local pulmonary inflammatory process, imbalance of pro-inflammatory/anti-inflammatory cytokines, exhaustion of the local pulmonary anti-oxidant system, augmentation of phosphodiesterase (PDE) activity, and provocation of epithelial/mesenchymal transition, which promotes the myofibroblast transformation, activation, and extracellular matrix deposition (2, 3, 5, 6). Unfortunately, there are no curable medications for PF, and the current medications aim to relieve symptoms and slow the progression of the disease. Also, these medications have limited efficacy, a high economic burden, many side effects, and cannot improve the mortality rate of PF (2, 4, 7). Therefore, we urgently need to develop a new medication that could show promise in treating PF. 

Recently, studies on PDE inhibitors have confirmed their antifibrotic potential in various animal models, such as renal and hepatic fibrosis, through the augmentation of tissue cAMP and/or cGMP levels, consequently inhibiting fibroblast/myofibroblast transformation, activation, and suppression of extracellular matrix deposition (8-10). Moreover, roflumilast (PDE4 inhibitor) and sildenafil (PDE5 inhibitor) exhibited antifibrotic effects in preclinical and clinical trials; however, they have many side effects that limit their use in PF (11). 

Vinpocetine, Ethyl-Apovincamine 22-oate, is one of the PDE-1 inhibitors that are synthetically obtained from the periwinkle plant alkaloid extract, vincamine. It is approved globally as a cerebroprotective agent to promote brain functions and is therefore indicated for the treatment of cognitive impairment, dementia, and stroke; as well, it is used as a brain enhancer supplementation (12). Recent studies have shown that vinpocetine has neuroprotective, cardioprotective, hepatoprotective, antithrombotic, anti-aging, and antifibrotic effects on the liver through its anti-oxidant, immunomodulatory, and anti-inﬂammatory activities (8, 12-14). In addition, it has been documented that vinpocetine inhibits rat cardiac myofibroblast activation and extracellular matrix protein synthesis in cardiac fibroblasts. Besides, vinpocetine is well tolerated for long-term therapy, with excellent safety and without toxicity in its therapeutic dose (12, 14). Therefore, in the present study, vinpocetine was examined for its antifibrotic potential in a rat model of PF induced by intratracheal administration of bleomycin concerning its effect on body weight gain rates, broncho-alveolar lavage fluid (BALF) total and differential cell count, cytokine levels, lactic dehydrogenase (LDH) activity, lung tissue PDE activity, oxidative stress indicators, hydroxyproline content, histopathological changes, fibrosis score, immunohistochemical expression of alpha-smooth muscle actin (α-SMA) and macrophage CD68. 

## Materials and Methods

***Drugs and chemicals***

The following drugs and chemicals were purchased commercially: vinpocetine, chloramine-T reagent, Ehrlich’s reagent, Türk solution, diaminobenzidine, and L-hydroxyproline (Sigma, St. Louis, MO, USA); phosphate-buffered saline (PBS), formalin buffered saline, hydroxide sodium (NaOH), citrate buffer, Giemsa, and hematoxylin & eosin stains (El Gomhuria Co., Tanta, Egypt); bleomycin (Cipla Ltd., Verna, Goa, India); thiopental sodium (EIPICO, Tenth of Ramadan City, Egypt); Mallory’s Trichrome stain (Rue des Suisses, Nanterre, France).

***Animals***

Male Wistar rats, aged nine weeks old, weighed 150-200 g, were used in the present study. The animals were brought from the Tanta Faculty of Medicine Animal House, Tanta, Egypt, and kept at room temperature in plastic cages, with free access to water and a standard laboratory diet. The animals were allowed to adapt for one week before the experimental conduction and exposed to a 12-hours (hr) light / dark cycle. The experiment was approved by the Research Ethics Committee of Experimental Animal Care and Use at Prince Sattam bin Abdulaziz University, Al-Kharj, Saudi Arabia (Approval No. SCBR-126-2021), adhering to its guidelines. All animal management was applied between 8:00 AM and 5:00 PM. 

***Experimental design***

Sixty Wistar rats were randomly distributed into six groups of ten rats each. Group I (CONT) consisted of normal rats injected intratracheally with 0.1 ml of PBS. Group II (BLE) was PF-induced rats by a single intratracheal injection of bleomycin 5 mg/kg dissolved in 0.1 ml PBS (15). Group III (VPL) was PF-induced rats treated orally with 5 mg/kg/day of vinpocetine dissolved in PBS. Group IV (VPM) was PF-induced rats treated orally with 10 mg/kg/day of vinpocetine dissolved in PBS. Finally, group V (VPH) was PF-induced rats treated orally with 20 mg/kg/day of vinpocetine dissolved in PBS (8). All treatments were freshly prepared daily and started immediately after the bleomycin instillation (day 1) through day twenty-one of the experiment. Twenty-four hours after the final treatment, rats were euthanized by intraperitoneal injection of 100 mg/kg thiopental sodium, then the BALF was collected, and the lungs were harvested. Then, the right lung was washed with ice-cold PBS, cut into small pieces, and homogenized. Half of the homogenate was centrifuged at 200 g for 10 min, and the supernatant was collected. The remaining half of the homogenate and the collected supernatant were stored at -80 ^°^C for further evaluation of the lung tissue protein level, PDE activity, oxidative stress indicators, and hydroxyproline content. The left lung was processed for histopathological and immunohistochemical studies.

***Evaluation of rats’ body weight gain rates***

Rats’ weight gain rate was evaluated by measuring animal weights before the experiment and just before animal euthanasia using Radwag balance (PS 510.R1, Poland). The body weight gain rate was assessed according to Balaha *et al.* using the following formula ((A-B)/B) (X 100), where A is the weight just before euthanasia and B is the weight before the experiment. The result was expressed as a percent (16).

***BALF collection and evaluation***

Immediately after animal euthanasia, BALF was collected, according to Balaha *et al.*, to evaluate the local lung inflammatory reaction by assessing BALF cytokine levels and inflammatory cell counts. Briefly, after animal euthanasia, the main left lung bronchus was ligated, the right lung was lavaged four times with 1.5 ml PBS, and nearly 5.4 ml of BALF was recovered per rat (90%) in a plastic tube and kept cool on ice. Later, the recovered BALF was centrifuged at 350 g for 10 min at 4 ^°^C, and the supernatant was collected and stored at -20 ^°^C for further analysis of BALF cytokine levels. Subsequently, each cell pellet was suspended in 1ml of PBS. Next, two drops of the suspension were mixed with Türk solution, and the total cell count was recorded in a hemocytometer chamber (Marienfeld, Improved Neubauer). The differential cell count was then recorded for 300 cells after staining with Giemsa stain and expressed as a percentage of the total cell count (17). 

***Evaluation of BALF cytokine levels***

BALF cytokine levels were assessed by evaluating BALF interleukin (IL)-6, IL-10, transforming growth factor beta 1 (TGF-β1), and tumor necrosis factor-alpha (TNF-α), using sandwich enzyme-linked immunosorbent assay (sELISA) kits according to the manufacturer protocols. All sELISA kits were purchased from CusaBio, Wuhan, Hubei, China, where the minimum detection limit of IL-6, IL-10, TGF-β1, and TNF-α were 0.078 pg/ml, 0.78 pg/ml, 1.56 pg/ml and 1.56 pg/ml, respectively. In addition, an automated plate reader (Stat Fax 2100, Fisher Bioblock Scientific, BP, Illkirch Cedex, France) was used to detect cytokine levels expressed as pg/ml BALF.

***Evaluation of BALF LDH activity***

The BALF LDH activity was evaluated using a colorimetric assay kit (Bio-Diagnostic Co., Dokki, Giza, Egypt) according to the manufacturer’s protocol, using the method described by Pesce (1987). The absorbance of the BALF samples was measured at a one-minute interval for 3 min using Biosystems semiautomated analyzer (BTS-350, Barcelona, Spain) at 340 nm. The BALF LDH activity was expressed as units per liter (U/L) (18).

***Evaluation of lung tissue protein level***

Lung tissue protein levels were evaluated consistently with the manufacturer protocol of a colorimetric assay kit (Bio-Diagnostic Co., Giza, Egypt), as described by Gornall *et al.,* where 0.025 ml of the lung tissue homogenate was added to 1.0 ml of Biuret reagent, appropriately mixed, then incubated for 10 min at 37 ^°^C. The optical densities were then measured at 550 nm. Results were expressed as mg lung tissue protein/ml lung tissue homogenate (19).

***Evaluation of lung tissue PDE activity***

According to the manufacturer protocol, Lung tissue PDE activity was evaluated using the sELISA kit purchased from Abcam (Waltham, MA, USA). The optical densities of the samples were assessed at 620 nm with an automatic microplate reader (Stat Fax 2100). The results were expressed as U/mg lung tissue protein, where each unit was equal to the hydrolysis of 1 nmol of 3’5’cAMP or 3’5’cGMP to 5’ AMP or 5’GMP respectively per minute under the conditions of the linearity assay (30 ^°^C, pH 7.4, 200 µM 3’5’ cAMP or cGMP).

***Evaluation of lung tissue oxidative stress indicators***

The lung tissue oxidative stress indicators were evaluated by assessing lung tissue malondialdehyde (MDA), reduced glutathione (GSH) levels, catalase (CAT), and superoxide dismutase (SOD) activities. The lung tissue MDA, GSH levels, CAT, and SOD activities were evaluated using colorimetric assay kits purchased from Bio-Diagnostic Co. (Dokki, Giza, Egypt), according to the manufacturer instructions, as described elsewhere (20-23). The lung tissue CAT and SOD activities were expressed as U/mg lung tissue protein, while the lung GSH and MDA levels were expressed as nmol/g lung tissue protein and nmol/mg lung tissue protein, respectively. All absorbances of lung oxidative stress indicators were evaluated using the Biosystems semiautomated analyzer (BTS-350) (20-23).

***Evaluation of lung tissue hydroxyproline content***

The lung tissue hydroxyproline content was evaluated as designated by Reddy and Enwemeka (24). Briefly, twenty-five μl of lung tissue homogenate was gently mixed with 25 μl NaOH (2 N final concentration), then autoclaved at 120 ^°^C for 20 min. Later, the mixture was held at room temperature for 25 min after adding 450 μl of chloramine-T reagent (0.056 M). Then, after adding and gently mixing 500 μl of Ehrlich’s reagent (1 M), the mixture was held for 20 min at 65 ^°^C. Finally, the sample absorbance was estimated after the chromophore’s development at 550 nm using Biosystems semiautomatic analyzer (BTS-350), and the hydroxyproline content in lung tissue was expressed as μg/g lung tissue’s protein on L-hydroxyproline standard curve. 

***Histopathological evaluations***

Lung tissues were processed according to Suvarna *et al.* (2018). Briefly, the samples were fixed in 10% formalin-buffered saline, embedded in hard paraffin, and cut into five µm thick sections. Later, sections were stained with hematoxylin and eosin stain (H&E) and Mallory’s Trichrome stain and examined under a light microscope (Olympus Optical Co., LTD, Japan) to assess histopathological and fibrotic changes in lung tissue (25). First, histopathological changes of lung tissue were graded regarding interalveolar septa thickening, interstitial mononuclear cell infiltrations, alveolar spaces collapse, bronchiolar epithelial lining proliferation, bronchiolar basal lamina detachment, and blood vessel wall thickening and congestion, using a 0-3 scale, where each parameter scored arbitrary as follow, 0=none, 1=mild, 2=moderate, and 3=sever changes. Then, the sections scored according to Hübner *et al.* for pulmonary fibrotic changes, where 0=no fibrosis (normal lung), 1=isolated alveolar septa with slight fibrotic changes, 2=fibrotic changes of the alveolar septa with knot-like formation, 3=contiguous fibrotic walls of the alveolar septa, 4=single fibrotic masses, 5=confluent fibrotic masses, 6=large contiguous fibrotic masses, 7=air bubbles, and 8=fibrous obliteration (26). 

***Immunohistochemical evaluation of lung tissue’s α-SMA and macrophage CD68 expression***

Immunohistochemical expressions of lung tissue α-SMA and CD68 were evaluated as described elsewhere (27, 28). Shortly, formalin-fixed, paraffin-embedded lung tissue specimens were deparaffinized, rehydrated, heated at the microwave for 20 min in citrate buffer (pH 6), and then washed with PBS. The sections were then treated with a blocking solution for 20 min, incubated with α-SMA primary antibody (1:800, Bio-techne, McKinley Place NE, Minneapolis, MN, USA), or macrophage CD68 primary antibody (1:400, BIO-RAD, Alfred Nobel Drive, Hercules, California, USA) for 30 or 60 min, respectively at room temperature, washed in PBS and incubated with secondary antibody for 10 min. Then, diaminobenzidine was added to each slide for 10 min, and the slides were counterstained with Mayer’s hematoxylin, dehydrated, and cleared. Finally, the sections were examined using a light microscope (Olympus Optical Co., LTD, Japan) (27, 28). Positive reaction to lung α-SMA myofibroblast and lung CD68 macrophages stained brown. A negative control section was obtained by replacing the primary antibody with PBS. The number of CD68-positive macrophages was counted manually, according to Pereira *et al.*, using an optical microscope (Olympus Optical Co., LTD, Japan). In summary, stained sections were screened for the areas of the highest intensity of CD68-positive macrophage expression at low power (X10), then macrophages from the manually selected fields were counted in ten high-power fields (HPF, X 40), and the macrophage average of the 10 HPF was calculated and expressed as macrophages / HPF (29). 

***Statistical analysis***

The statistical package for the Social Sciences Statistics Software for Windows (IBM-SPSS, Version 25, Armonk, New York, USA) was used to analyze the present study’s data, and the results were expressed as mean±SD. The data of all groups were compared with a one-way analysis of variance (ANOVA), followed by the Bonferroni test as a *post-hoc* test. *P*-value < 0.05 is considered significant.

**Table 1:** Effect of vinpocetine on rats’ body weight gain rates

**Group**	**Before experiment (g)**	**After experiment (g)**	**Body weight gain rate (%)**
CON	181.65 ±‎ 19.12‎	222.83 ±‎ ‎20.63‎	13.48 ±‎ 2.31‎‎
BLE	185.9‎ ±‎ ‎17.92‎	192.1 ±‎ ‎14.45‎	3.65 ±‎ ‎1.54‎^*^^*^^*^
VPL	182.8‎ ±‎ ‎20.24‎	195.61 ±‎ ‎19.36‎	6.73 ±‎ 1.6‎ ^+^^+^
VPM	185.44±‎15.98	212.47 ±‎ ‎15.61‎	8.56 ±‎ ‎1.9‎ ^+^^#^
VPH	180.92±‎20.04	219.43 ±‎ ‎14.38‎	13.2 ±‎ ‎2.03‎ ^###^

***P<0.001 (Vs. CON group), ^#^*P<*0.05 & ^###^*P<*0.001 (Vs. BLE group), ^+^*P<*0.05 & ^++^*P<*0.01 (Vs. VPH group)

Results are expressed as mean±SD

CON:control group, BLE, bleomycin-induced pulmonary fibrosis group, VPL: pulmonary fibrosis group treated by vinpocetine 5 mg/kg/day, VPM: pulmonary fibrosis group treated by vinpocetine 10 mg/kg/day, VPH: pulmonary fibrosis group treated by vinpocetine 20 mg/kg/day

**Figure 1:**
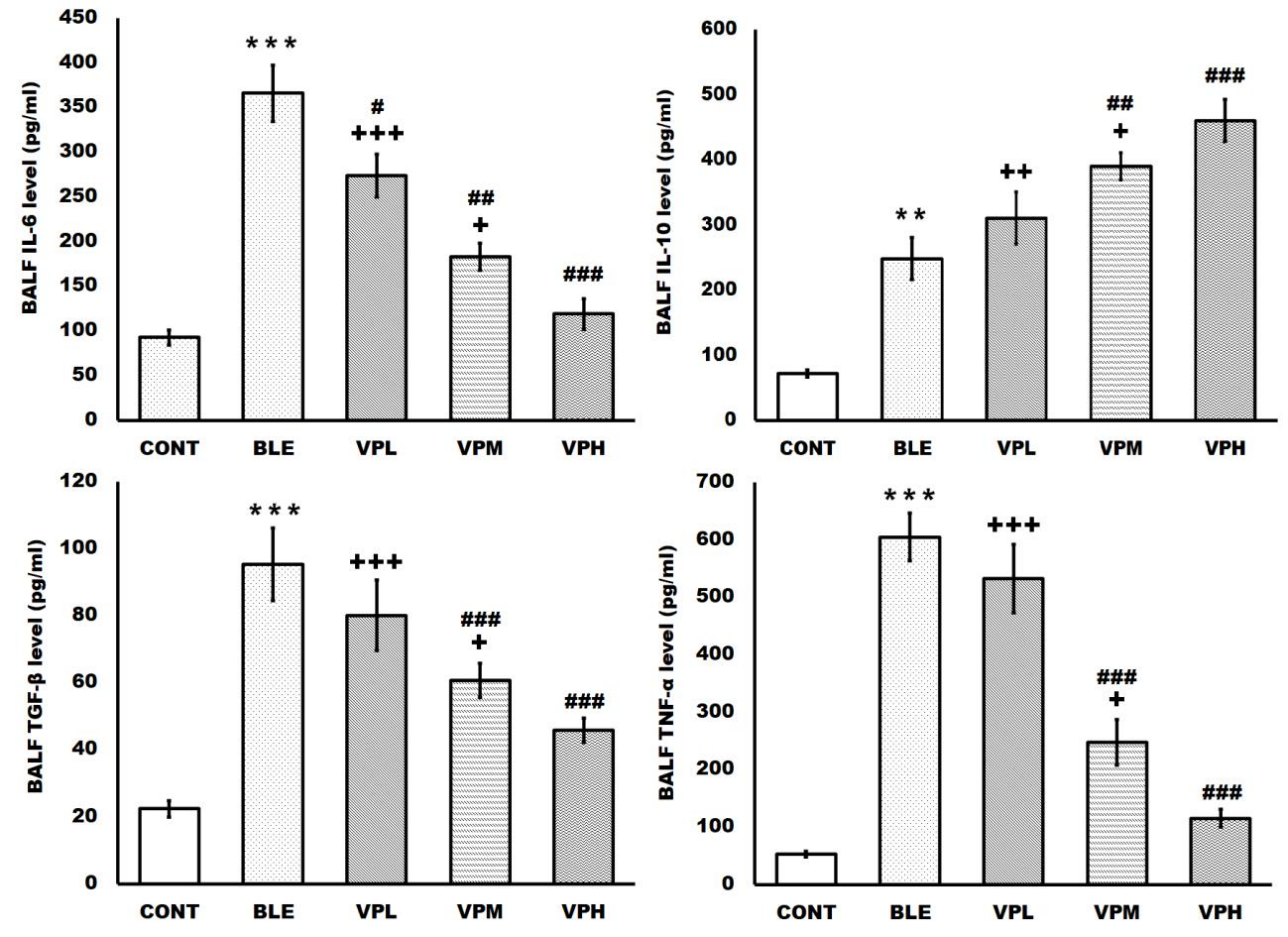
Effect of vinpocetine on broncho-alveolar lavage fluid (BALF) cytokine levels

Results are expressed as mean±SD

**P<0.01 & ***P<0.001 (Vs. CON group), ^#^*P<*0.05, ^##^*P<*0.01 & ^###^*P<*0.001 (Vs. BLE group), ^+^*P<*0.05, ^++^*P<*0.01 & ^+++^*P<*0.001 (Vs. VPH group)

CON: control group, BLE: bleomycin-induced pulmonary fibrosis group, VPL: pulmonary fibrosis group treated by vinpocetine 5 mg/kg/day, VPM: pulmonary fibrosis group treated by vinpocetine 10 mg/kg/day, VPH: pulmonary fibrosis group treated by vinpocetine 20 mg/kg/day

**Table 2:** Effect of vinpocetine on BALF total and differential cell count

**Group**	**Total cell count** **(10** ^5^ **/ml BALF)**	**Macrophage** **%**	**Lymphocyte** ** %**	**Neutrophils ** **%**
CON	0.51 ± 0.11	97.02 ± 0.42	2.08 ± 0.31	0.9 ± 0.11
BLE	4.31 ± 1.16^***^	65.34 ± 7.89^***^	20.38 ± 2.98^***^	14.28 ± 1.96^***^
VPL	3.65 ± 1.89^+++^	72.47 ± 5.98^+++^	18.34 ± 1.95^+++^	9.19 ± 2.93^++^
VPM	1.99 ± 0.31^##+^	81.72 ± 2.87^##+^	12.45 ± 1.34^##++^	6.74 ± 0.73^#+^
VPH	1.12 ± 0.23^###^	88.56 ± 3.39^###^	7.32 ± 0.99^###^	5.12 ± 0.65^###^

*** *P<*0.001 (Vs. CON group), ^#^*P<*0.05, ^##^*P<*0.01 & ^###^*P<*0.001 (Vs. BLE group), ^+^*P<*0.05, ^++^*P<*0.01 & ^+++^*P<*0.001 (Vs. VPH group)

Results are expressed as mean±SD

CON: control group, BLE: bleomycin-induced pulmonary fibrosis group, VPL; pulmonary fibrosis group treated by vinpocetine 5 mg/kg/day, VPM: pulmonary fibrosis group treated by vinpocetine 10 mg/kg/day, VPH: pulmonary fibrosis group treated by vinpocetine 20 mg/kg/day

**Figure 2:**
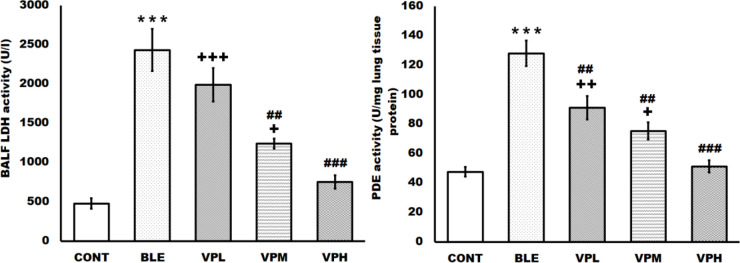
Effect of vinpocetine on broncho-alveolar lavage fluid (BALF), lactic dehydrogenase (LDH) and lung tissue phosphodiesterase (PDE) activities

Results are expressed as mean±SD

***P<0.001 (Vs. CON group), ^#^*P<*0.05, ^##^P<0.01 & ^###^*P<*0.001 (Vs. BLE group), ^+^*P<*0.05, ^++^*P<*0.01 & ^+++^*P<*0.001 (Vs. VPH group)

CON: control group, BLE: bleomycin-induced pulmonary fibrosis group, VPL: pulmonary fibrosis group treated by vinpocetine 5 mg/kg/day, VPM: pulmonary fibrosis group treated by vinpocetine 10 mg/kg/day, VPH: pulmonary fibrosis group treated by vinpocetine 20 mg/kg/day

**Figure 3:**
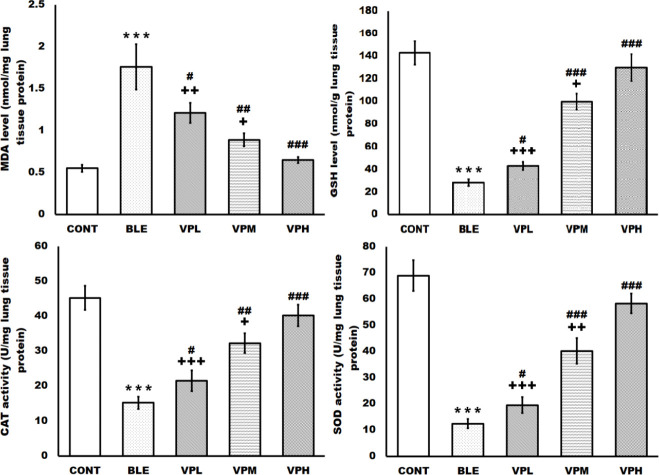
Effect of vinpocetine on lung tissue oxidative stress indicators

Results are expressed as mean±SD

***P<0.001 (Vs. CON group), ^#^*P<*0.05, ^##^*P<*0.01 & ^###^*P<*0.001 (Vs. BLE group), ^+^*P<*0.05, ^++^*P<*0.01 & ^+++^*P<*0.001 (Vs. VPH group)

CON: control group, BLE: bleomycin-induced pulmonary fibrosis group, VPL: pulmonary fibrosis group treated by vinpocetine 5 mg/kg/day, VPM: pulmonary fibrosis group treated by vinpocetine 10 mg/kg/day, VPH: pulmonary fibrosis group treated by vinpocetine 20 mg/kg/day

**Figure 4:**
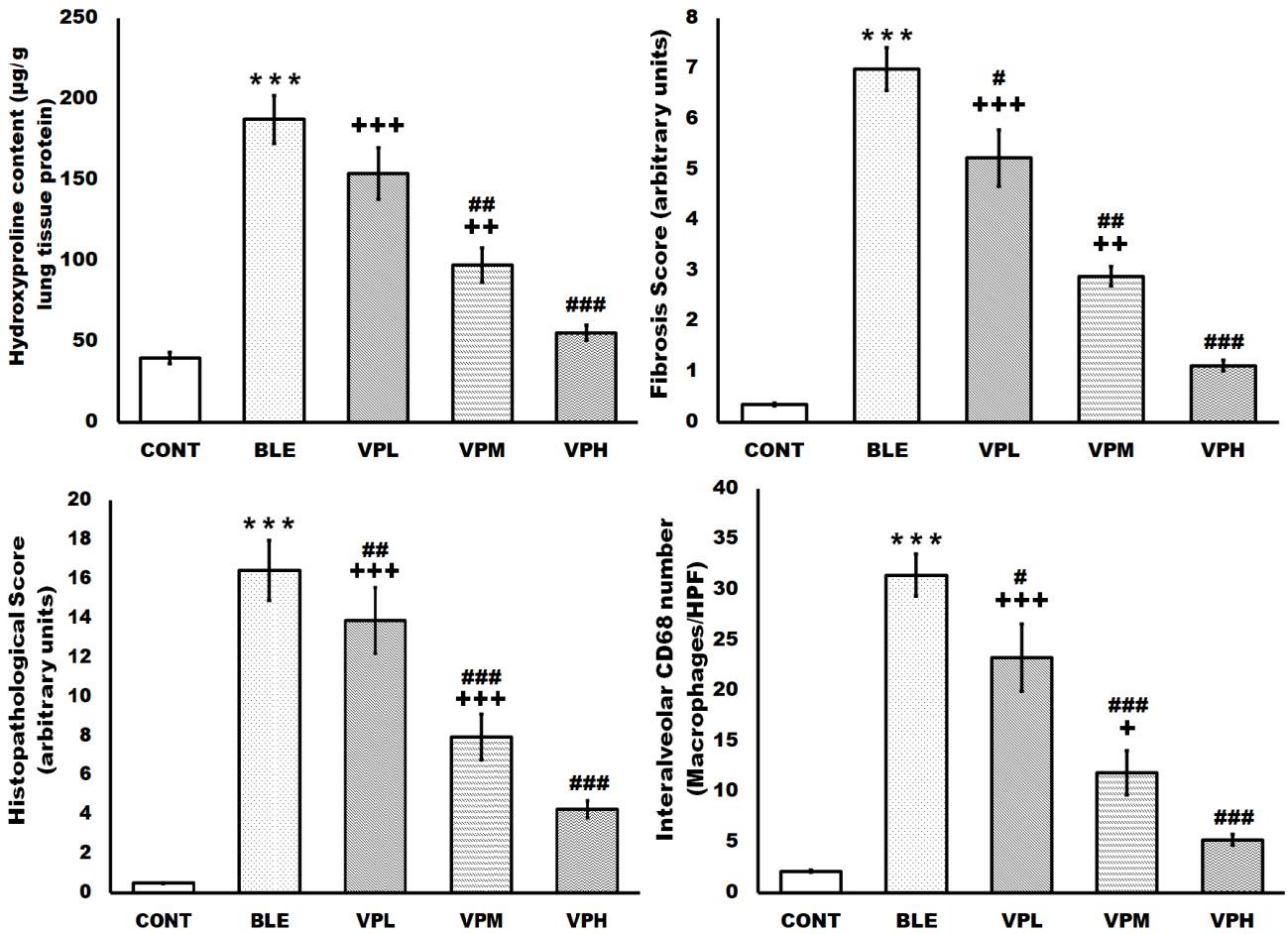
Effect of vinpocetine on lung tissue hydroxyproline content, fibrosis score, and immunohistochemical CD68 macrophage expression

Results are expressed as mean±SD

***P<0.001 (Vs. CON group), ^#^*P<*0.05, ^##^*P<*0.01 & ^###^P<0.001 (Vs. BLE group), ^+^*P<*0.05, ^++^*P<*0.01 & ^+++^*P<*0.001 (Vs. VPH group)

CON: control group, BLE: bleomycin-induced pulmonary fibrosis group, VPL: pulmonary fibrosis group treated by vinpocetine 5 mg/kg/day, VPM: pulmonary fibrosis group treated by vinpocetine 10 mg/kg/day, VPH: pulmonary fibrosis group treated by vinpocetine 20 mg/kg/day

**Figure 5:**
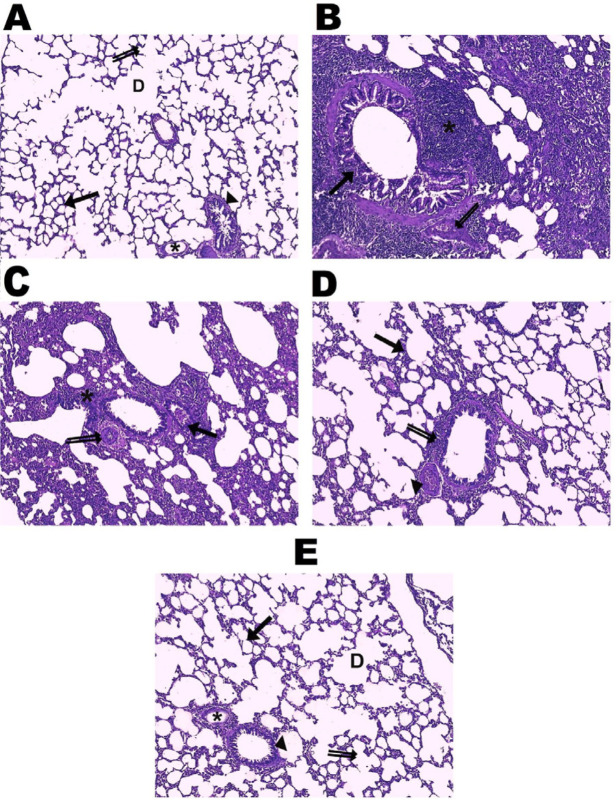
Effect of vinpocetine on H&E stained sections. A, the control group showed numerous alveoli (→), alveolar sacs (double arrow), alveolar ducts (D), respiratory bronchioles lined with ‎simple columnar ciliated epithelium, smooth muscle, and connective tissue (►) and normal blood vessels (*). B, the BLE group showed thickened interalveolar septa with massive interstitial mononuclear cellular infiltrations (*), disorganized, proliferative bronchioles’ lining epithelium with detached parts from its basal lamina (→), and congested blood vessels with a thickened wall (double arrow). C, the VPL group showed interstitial mononuclear cellular infiltrations ‎‎(*), disorganized bronchioles’ lining epithelium (→), and thickened ‎blood vessels’ wall (double arrow). D, the VPM group showed ‎thickened interalveolar septa (→), peribronchiolar mononuclear cellular ‎infiltrations (double arrow), and congested blood vessels (►). E, the VPH group showed nearly normal alveoli (→), alveolar sacs (double arrow), alveolar ducts (D), blood vessels (*), and respiratory bronchioles (►), (H&E, X 100)

**Figure 6:**
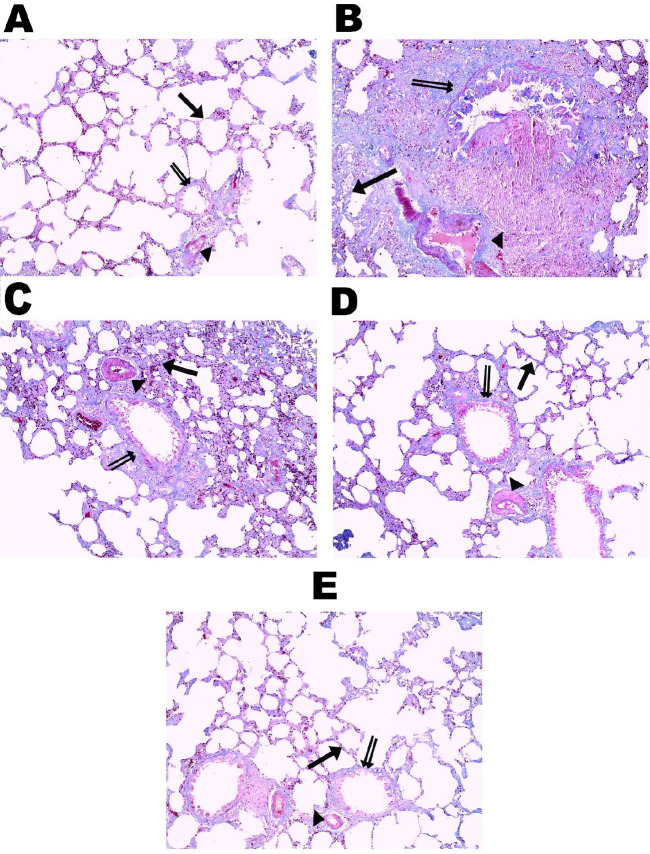
Effect of vinpocetine on Mallory’s trichrome stained sections. A, the control group showed little collagen fibers in the interalveolar ‎septa (→), wall of blood capillaries (►), and bronchioles (double arrow). B, the BLE group showed increased deposition of collagen fibers in the interalveolar septa (→), blood capillaries’ wall (►), and bronchioles (double arrow) with marked thickening of their walls. C, the VPL group showed increased deposition of collagen fibers in the interalveolar septa (→), wall of blood ‎capillaries (►), and bronchioles (double arrow). D, the VPM showed a moderate amount of collagen fibers in the interalveolar septa (→), wall of blood capillaries (►), and bronchioles (double arrow). E, the VPH group showed little collagen fibers in the interalveolar septa (→), in the wall of blood capillaries (►) and bronchioles (double arrow), (Mallory’s trichrome, X100)

**Figure 7:**
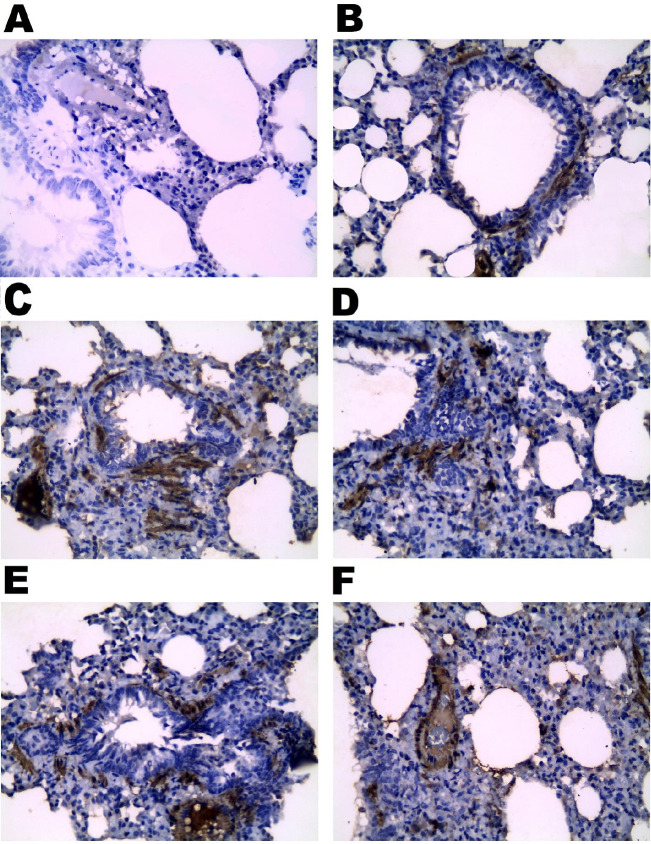
Effect of vinpocetine on α-SMA immunohistochemical expression. A, negative control showed no immunohistochemical reaction, B, positive control showed that the expression of α-SMA was restricted to the alveolar and the blood vessel walls of the lungs. C, BLE group showed extensive α-SMA expression in the lung parenchyma. D, the VPL group showed moderate α-SMA expression in the lung parenchyma. E, the VPM group showed low α-SMA expression in the lung parenchyma. F, VPH group showed α-SMA immunohistochemical ‎expression in the alveolar and blood vessels’ wall of the lung parenchyma, like the control group, (α-SMA, X 400)

**Figure 8:**
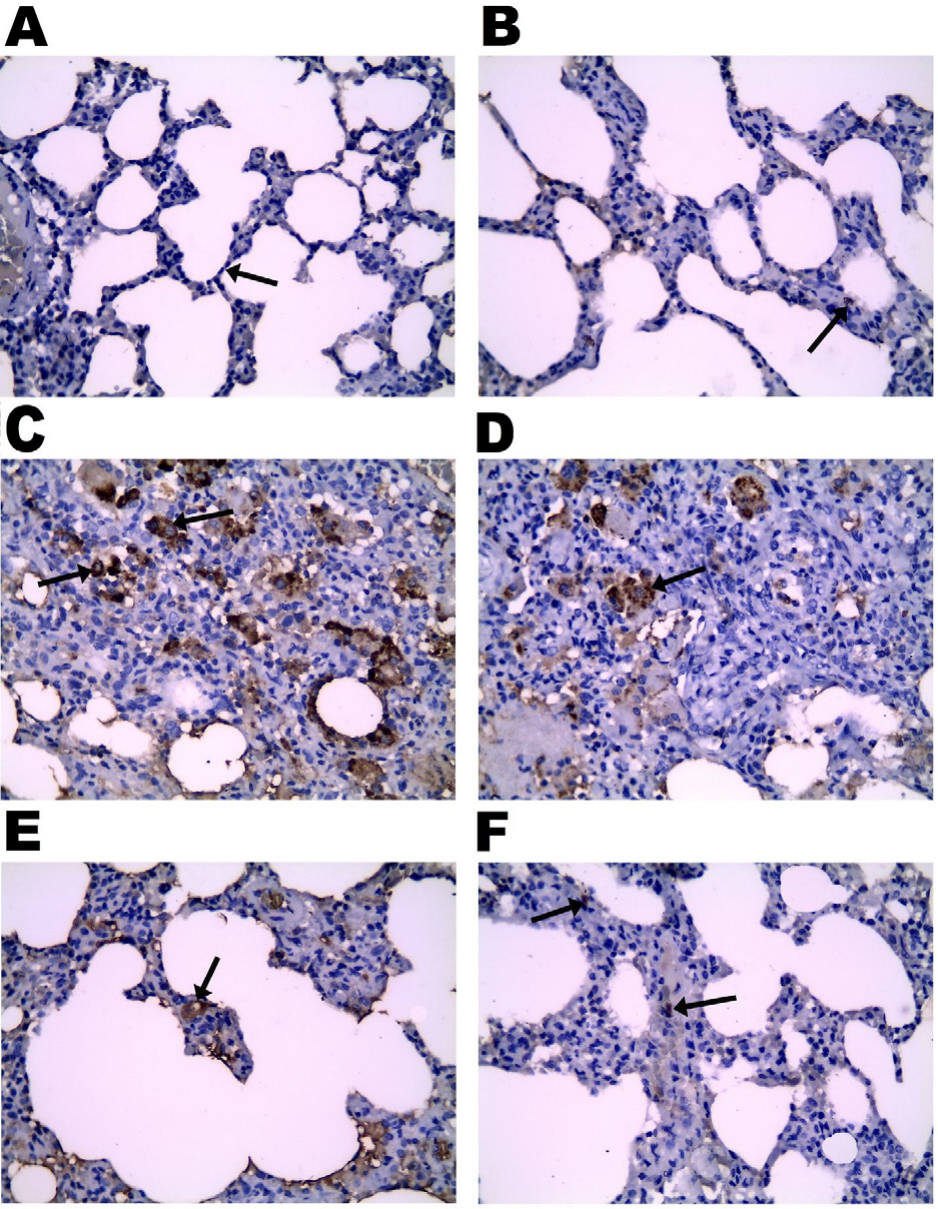
Effect of vinpocetine on CD68 macrophages immunohistochemical expression. A, negative control showed no immunohistochemical reaction (→). B, positive control showed few positively stained macrophages in the lung interalveolar septa (→). C, the BLE group showed numerous positively stained brown macrophages in the interalveolar septa (→). D & E, VPL & VPM groups with a moderate number of brown positively stained macrophages (→). F, VPH group expressed few brown positively stained macrophages in a picture near to the control (→), (CD68 macrophages, X 400)

## Results

***Effect of vinpocetine on rats’ body weight gain rates***

Bleomycin administration significantly reduced the rate of weight gain in rats compared to the control group. In addition, the vinpocetine treatment significantly prevented the reduction in the rate of weight gain in rats, in a dose-dependent manner, compared to the BLE group. The VPH group showed a more significant elevation of the decreased rate of weight gain in rats compared to the VPM and the VPL groups. Furthermore, no significant differences were found between the BLE and VPL groups (Table 1). 

***Effect of vinpocetine on BALF total and differential cell count***

Intratracheal administration of bleomycin enhanced the local pulmonary inflammatory cell response, as indicated by a significant rise in BALF total cell count, the percentage of lymphocytes and neutrophils, and a significant reduction in the percentage of macrophages. However, vinpocetine treatment suppressed the local pulmonary inflammatory cell reaction, as it effectively suppressed the increased total BALF cell count and the percentage of lymphocytes and neutrophils; meanwhile, the percentage of reduced macrophages increased significantly in a dose-dependent manner. The VPH group showed more significant suppression of the increased total BALF cell count, the percentage of lymphocytes and neutrophils, and a more significant elevation of the reduced macrophage percentage compared to the VPM and the VPL groups (Table 2).

***Effect of vinpocetine on BALF cytokine levels***

Simultaneously with the suppression of the pulmonary inflammatory cellular response, treatment with vinpocetine significantly and dose-dependently reduced the increased levels of BALF pro-inflammatory cytokines and significantly augmented the production of the anti-inflammatory cytokine induced by intratracheal administration of bleomycin. As a result, the significantly elevated BALF IL-6, TGF-β1, and TNF-α levels were significantly suppressed. Meanwhile, the elevated BALF IL-10 level was significantly augmented by the vinpocetine treatment since the VPH group showed more significant suppression of the raised BALF IL-6, TGF-β1, and TNF-α levels and more significant augmentation of the raised BALF IL-10 compared to the VPM and the VPL groups. In addition, the VPL group showed a significant decrease in the elevated BALF IL-6 level, which was increased by the bleomycin intratracheal administration (Figure 1).

***Effect of vinpocetine on BALF LDH and lung tissue PDE activities***

The BLE group showed significant augmentation of the BALF LDH and lung tissue PDE activities compared to the CONT group. On the other hand, vinpocetine treatment significantly reduced the elevated BALF LDH and lung tissue PDE activities in a dose-dependent manner. The VPH group showed a more significant reduction in elevated BALF LDH and lung tissue PDE activities than the VPM and VPL groups. Moreover, the VPL group showed a significant decrease in the lung tissue’s PDE activity compared to the BLE group (Figure 2).

***Effect of vinpocetine on lung tissue oxidative stress indicators***

The vinpocetine treatment dose-dependently restored the depleted anti-oxidant system of the lung tissues, which was disturbed with bleomycin intratracheal administration. Compared to the control group, intratracheal bleomycin administration increased the MDA and reduced the lung tissue’s GSH levels, CAT, and SOD activities. However, vinpocetine treatment dose-dependently and significantly reduced the elevated MDA and significantly increased the reduced GSH levels, CAT, and SOD activities in lung tissue compared to the BLE group. The VPH group demonstrated a more significant reduction of the elevated MDA level and a more significant enhancement of the reduced GSH level, CAT, and SOD activities in the lung tissue compared to the VPM and the VPL groups (Figure 3). 

***Effect of vinpocetine on lung tissue hydroxyproline content***

The hydroxyproline content of lung tissue increased significantly after a single intratracheal administration of bleomycin compared to the CONT group. Conversely, the treatment with vinpocetine suppressed the increased hydroxyproline content in lung tissue induced by intratracheal administration of bleomycin in a dose-dependent manner. The VPH group showed a more significant reduction in the increased hydroxyproline content of the lung tissue compared to the VPM and the VPL groups (Figure 4).

***Effect of vinpocetine on lung tissue histopathological changes and fibrosis score***

*H* *&* *E results*

The sections of the control group stained with H&E showed lung tissue with alveoli separated by a thin interalveolar connective tissue septum and blood capillaries lined with a simple squamous endothelial and basal lamina. Also, alveolar sacs, alveolar ducts, and respiratory bronchioles were observed. The bronchioles were different in size, lined with simple columnar ciliated epithelium, smooth muscle, and connective tissue. However, the BLE group exhibited abnormal lung morphology, thickened interalveolar septa, massive interstitial mononuclear cellular infiltrations, and collapsed alveolar spaces. In addition, the epithelium lining of the bronchioles appeared disorganized and proliferative, with detached parts of its basal lamina and congested thickened wall blood vessels. Meanwhile, the vinpocetine treatments showed dose-dependent protection compared to bleomycin-induced lung injury. The VPH-treated group revealed that most of the lung tissue resembled the control group, in contrast to the thickened interalveolar septa, peribronchiolar mononuclear cellular infiltrations, and congested blood vessels in the VPM group, and the persistence of most of the lung tissue pathology in the VPL group.

Besides these findings, the BLE group had a significant increase in pathological score compared to the control group, which was significantly and dose-dependently amended by vinpocetine treatment. In contrast, the VPH group showed a more efficient reduction of the pathological score than the VPL and VPM groups (Figures 4 & 5).

*Mallory’s trichrome results*

The Mallory’s trichrome-stained sections of the control group revealed little collagen fibers in the interalveolar septa as well as in the wall of blood capillaries and bronchioles, in contrast to the BLE group, which showed an increased deposition of the interalveolar septa, the capillaries, and bronchioles walls’ collagen fibers, with marked bronchiolar wall thickening. The VPH group showed an almost normal picture like the control group, in contrast to the VPL and VPM groups, which showed severe-moderate and moderate-mild deposition of the collagen fibers, respectively (Figure 6).

***Fibrosis score***

Compared to the control group, the BLE group showed a significant increase in fibrotic changes in the interalveolar septa, the wall of the blood capillaries, and the bronchioles. In vinpocetine-treated groups, collagen fiber deposition was significantly reduced dose-dependently compared to the BLE group. The VPH group showed a more significant reduction in the lung tissue fibrosis score compared to the VPM and VPL groups. In addition, the VPL group revealed a significant reduction in the lung tissue fibrosis score compared to the BLE group (Figure 4).

***Effect of vinpocetine on lung tissue’s α-SMA and macrophage CD68 immunohistochemical expression***

*Immunohistochemical results of lung α-SMA*

The immunohistochemical expression of α-SMA in the control group was restricted to the lungs’ alveolar and blood vessel walls. In contrast, the BLE group showed extensive myofibroblast α-SMA expression in the lung parenchyma instead of the moderate and low expressions observed in the VPL and VPM groups, respectively. In addition, the immunohistochemical expression of the α-SMA of the VPH group was observed in the alveolar and blood vessels’ wall of the lung parenchyma, which was like that of the control group (Figure 7). 

*CD68 immunohistochemical expression of lung tissue macrophage*

The control group showed a few macrophages in the interalveolar septa of the lung that appeared as positively stained brown cells, while the BLE group had numerous positively stained brown macrophages in the interalveolar septa, in contrast to the modest increase in the VPL and VPM treated groups. On the other hand, the VPH-treated group expressed a few brown positively stained macrophages in a picture like the control group (Figure 8). 

Statistically, the BLE group showed a significant increase in CD68-positive macrophages compared to the control group. However, there was a dose-dependent reduction in the number of CD68-positive macrophages compared to the BLE group in vinpocetine-treated groups since the VPH group showed a more significant reduction in the number of CD68-positive macrophages compared to the VPM and VPL groups. Furthermore, the VPL group showed a significant reduction in CD68-positive macrophages compared to the BLE group (Figure 4).

## Discussion

PF is a global health problem, affecting 8-60 per 100,000 cases per year worldwide, with an incidence rising to 200 per 100,000 cases per year after the age of 70 and increasing annually, with a high economic burden (1). Several animal models have been used to induce PF to explain the mechanisms of PF development and to explore the potentiality of the new therapeutic agents. Bleomycin-induced PF is one of the most widely used and best-studied models for the induction of PF (30, 31). Bleomycin is a cytotoxic antibiotic obtained from *Streptomyces verticillatus* and used to treat many types of cancers; however, the main restrictive disadvantage of bleomycin is the induction of lung injury that ultimately ends in PF (32). Bleomycin induces PF through DNA breakdown and prevents its repair through the formation of excessive reactive oxygen species (ROS), and lung tissue contains small amounts of bleomycin hydrolase, which is why bleomycin induces severe lung injury and inflammation, with PF is the final consequence (30). 

Intratracheal administration of bleomycin is the best model resembling the pathogenesis of PF in humans since bleomycin induces the characteristics of PF seen in humans, including fibroblasts proliferation and differentiation and the deposition of excessive extracellular matrix proteins, with the destruction of the alveolar architecture (30, 33). In addition, bleomycin produces an excessive amount of ROS, which induces an inflammatory reaction similar to what happened in PF in humans, as it promotes the secretion of a massive amount of the pro-inflammatory cytokines such as IL-6 and TNF-α, with subsequent secretion of the profibrotic cytokines such as TGF-β1, and the profibrotic markers such as type 1 collagen and α-SMA (30, 31, 34). Furthermore, once bleomycin is administrated intratracheally, it leads to severe lung damage and inflammation with subsequent induction of fibrosis within a short time. Fibrotic reactions start seven days after intratracheal administration of bleomycin, and the fibrosis becomes histopathologically evident on day 14 after intratracheal administration of bleomycin. However, the maximal fibrotic response was found on day 21 after intratracheal administration of bleomycin (34, 35). 

Recent studies have shown that vinpocetine has neuroprotective, cardioprotective, hepatoprotective, antithrombotic, anti-aging, and antifibrotic effects on the liver through its anti-oxidant, immunomodulatory, and anti-inﬂammatory activities (8, 12-14). In addition, it has been documented that vinpocetine inhibits rat cardiac myofibroblast activation and extracellular matrix protein synthesis in cardiac fibroblasts (14). Besides, vinpocetine is well tolerated for long-term therapy, with excellent safety and without toxicity in its therapeutic dose (12, 14). Therefore, vinpocetine was examined for its antifibrotic potentiality in the present study concerning its effect on rates of body weight gain, BALF total and differential cell count, cytokine levels, LDH, and lung tissue PDE activities, oxidative stress indicators, hydroxyproline content, histopathological changes, fibrosis score, and immunohistochemical expression α-SMA and macrophage CD68, in a rat model of PF induced by intratracheal administration of bleomycin. 

The data from the present study showed that vinpocetine is effective in a dose-dependent manner in ameliorating the PF induced by the intratracheal administration of bleomycin. Vinpocetine restored the reduced weight gain rates induced by bleomycin in rats. In addition, it suppressed the local pulmonary inflammatory cell reaction since it effectively suppressed the increased total BALF cell count and the percentage of lymphocytes and neutrophils. Meanwhile, the percentage of reduced macrophages was increased efficiently, the levels of pro-inflammatory and profibrotic BALF cytokines such as IL-6, TNF-α, and TGF-β1 was decreased, and the production of the anti-inflammatory cytokine IL-10 was significantly augmented. In the meantime, it restored the normal pulmonary architecture and reduced the elevated CD68-positive macrophage expression.

Moreover, vinpocetine restored the depleted anti-oxidant system of the lung tissue, as it effectively reduced the elevated MDA and increased the reduced GSH levels, CAT, and SOD activities in the lung tissue. Additionally, it decreased the augmented activities of BALF LDH and the lung tissue PDE. Furthermore, it reduced the increase in collagen deposition in the lung parenchyma, as it reduced the elevated levels of hydroxyproline in the lung tissue and the fibrosis score and restored the normal pulmonary collagen fiber distribution, as shown in Mallory’s trichrome stained sections and immunohistochemical expression of the lung parenchymal α-SMA.

The Animal weight gain rate is one of the commonly used methods to assess the animal’s health status during the progression of bleomycin-induced PF (36). Bleomycin administration in the present study decreased the rats’ weight gain rate throughout the experiment. Comparable results have been reported in previous studies; for example, a decrease in body weight of the animals reported after bleomycin administration, which was explained by a decrease in muscle mass of the animals and dysfunction of the gastrointestinal and pulmonary system after the administration of bleomycin (36, 37). However, vinpocetine treatment in the present study restored the normal weight gain rate in a dose-dependent manner. Consistent with our data, other researchers documented that the vinpocetine treatment prevented weight loss in animals in a rat model of hepatic fibrosis and in a guinea pig model of hearing loss (8, 38). 

Depleting the pulmonary anti-oxidant system is believed to be one of the most important molecular mechanisms that initiate and propagate the pulmonary fibrotic response following intratracheal administration of bleomycin (15, 39). Reducing anti-oxidant agents such as GSH, anti-oxidant enzymes such as SOD and CAT, and the accumulation of end products of oxidative stress such as MDA are the primary pulmonary oxidative stress indicators (2). Oxidative stress, in turn, promotes pro-inflammatory and profibrotic cytokines secretion, M2 macrophage polarization, myofibroblast differentiation and activation, extracellular matrix deposition, and apoptosis (6). In addition, oxidative stress promotes inflammation by recruiting inflammatory cells such as macrophages and neutrophils, which augment the oxidative stress with further propagation and chronicity of the inflammatory response and initiation of the lung parenchyma fibrotic reaction (15). Furthermore, increased LDH activity is a fundamental marker of cellular damage, interstitial deterioration, pulmonary parenchymal injury, and fibrosis due to oxidative stress (39). The data of the present study showed that vinpocetine dose-dependently restored the anti-oxidant system of the lung parenchyma, which is depleted by intratracheal administration of bleomycin, depending on the effective reduction of the elevated MDA level, and the effective elevation of the reduced GSH level, CAT, and SOD activities in the lung tissue. Matching these data, other researchers reported that vinpocetine efficiently reduced the raised striatal MDA level and enhanced the decreased striatal total anti-oxidant and SOD activities in a rat model of neurotoxicity. Also, it has been documented that vinpocetine effectively reduced raised hepatic MDA and NO levels and LDH activity and enhanced decreased hepatic GSH levels in a rat model of hepatic fibrosis (8, 13).

Chronic pulmonary inflammation is currently considered an essential contributing factor to the development and progression of PF. The augmented secretion of many growth factors, chemokines, and cytokines during chronic pulmonary inflammation thus promoted fibroblast/myofibroblast differentiation, proliferation, activation, and extracellular matrix deposition, with the development of fibrotic foci propagating into total PF (4, 30). TGF-β1 is one of the vital profibrotic cytokines involved in the development and progression of PF. It enhances the recruitment of inflammatory cells, including macrophages and neutrophils, into the lung parenchyma by promoting the secretion of pro-inflammatory cytokines such as TNF-α and IL-6 and by inhibiting the secretion of anti-inflammatory cytokines such as IL-10, sequentially stimulating fibroblast/myofibroblast transformation, myofibroblast activation, and extracellular matrix protein deposition such as collagen and α-SMA (4, 40, 41). In the present study, vinpocetine reduced the increased levels of BALF pro-inflammatory and profibrotic cytokines, IL-6, TNF-α, and TGF-β1 levels, and significantly augmented the production of the anti-inflammatory cytokine, IL-10. Similarly, it has been recorded that vinpocetine efficiently reduced increased levels of hepatic IL-6, TNF-α, and TGF-β1, in a rat model of hepatic fibrosis. Also, it has been demonstrated that vinpocetine suppressed elevated brain levels of IL-6 and TNF-α, and augmented brain level of IL-10, in a rat model of autism spectrum disorder (8, 42).

The enhancement of PDE1 expression and activity in the lung parenchyma is believed to be one of the crucial mechanisms of PF development and progression by promoting TGFβ1 secretion through cAMP and cGMP depletion, which leads to fibroblast/myofibroblast transformation, myofibroblast activation, and extracellular matrix deposition ultimately ending in PF (11, 43). Vinpocetine treatment in the present study dose-repressed the raised activity of PDE induced by the intratracheal administration of bleomycin. In harmony with the present study data, another study recorded that vinpocetine could suppress hepatic fibrosis induced by diethylnitrosamine (DEN) by inhibiting PDE activity and augmenting hepatic tissue cAMP levels (44).

PF induced by intratracheal bleomycin administration is characterized by continuous fibroblasts/myofibroblast transformation, myofibroblast activation, and excessive collagen production and deposition, with interstitial fibrosis and scarring of the lung parenchyma (39). In the present study, bleomycin intratracheal administration induced severe PF, as shown by Mallory’s trichrome stained sections, the fibrosis score, the elevated lung parenchymal hydroxyproline content, and the increased immunohistochemical expression of α-SMA, with the recruitment of CD68 positive macrophages into the lung parenchyma. On the other hand, the treatment with vinpocetine decreased lung parenchymal extracellular matrix deposition, which was reflected in the decrease of the elevated lung parenchymal hydroxyproline content, the fibrosis score, Mallory’s trichrome stained sections, and the immunohistochemical α-SMA expression, as well as a decrease in CD68 positive macrophages expression. In agreement with the data of the present study, other reports have demonstrated that vinpocetine treatment ameliorates hepatic fibrosis in rats with an effective reduction in hepatic collagen deposition, hydroxyproline content, and α-SMA immunohistochemical expression (8, 44). In addition, it has been documented that vinpocetine inhibits the rat cardiac myofibroblast activation and extracellular matrix protein synthesis in cardiac fibroblasts (14). 

## Conclusion

 The present study data disclosed that vinpocetine could ameliorate PF induced by intratracheal bleomycin administration in rats via its anti-oxidant, anti-inflammatory, antifibrotic, and PDE-inhibiting activities, thereby restoring the normal pulmonary architecture and function. Therefore, vinpocetine may propose a new promising agent in PF management.

## Authors’ Contributions

MB and AA Designed the experiments; MB, AA, SK and MB Performed experiments and collected data; MB, AA, SK and MB Discussed the results; MB, AA, SK and MB Analyzed and interpreted the results; MB Supervised, directed and managed the study; MB, AA, SK and MB Final approved of the version to be published.

## Conflicts of Interest

The authors declare that there were no conflicts of interest.

## References

[1] Nalysnyk L, Cid-Ruzafa J, Rotella P, Esser D (2012). Incidence and prevalence of idiopathic pulmonary fibrosis: Review of the literature. Eur Respir Rev.

[2] Wang L, Li S, Yao Y, Yin W, Ye T (2021). The role of natural products in the prevention and treatment of pulmonary fibrosis: A review. Food Func.

[3] Zhang Y, Gu L, Xia Q, Tian L, Qi J, Cao M (2020). Radix astragali and radix angelicae sinensis in the treatment of idiopathic pulmonary fibrosis: a systematic review and meta-analysis. Front Pharmacol.

[4] Phan THG, Paliogiannis P, Nasrallah GK, Giordo R, Eid AH, Fois AG (2021). Emerging cellular and molecular determinants of idiopathic pulmonary fibrosis. Cell Mol Life Sci.

[5] Huang Y-Y, Deng J, Tian Y-J, Liang J, Xie X, Huang Y (2021). Mangostanin derivatives as novel and orally active phosphodiesterase 4 inhibitors for the treatment of idiopathic pulmonary fibrosis with improved safety. J Med Chem.

[6] Kseibati MO, Shehatou GS, Sharawy MH, Eladl AE, Salem HA (2020). Nicorandil ameliorates bleomycin-induced pulmonary fibrosis in rats through modulating eNOS, iNOS, TXNIP and HIF-1α levels. Life Sci.

[7] Chen R-r, Li Y-j, Chen J-j, Lu C-l (2020). A review for natural polysaccharides with anti-pulmonary fibrosis properties, which may benefit to patients infected by 2019-nCoV. Carbohydr Polym.

[8] Elnfarawy AA, Nashy AE, Abozaid AM, Komber IF, Elweshahy RH, Abdelrahman RS (2021). Vinpocetine attenuates thioacetamide-induced liver fibrosis in rats. Hum Exp Toxicol.

[9] Xu M, Li S, Wang J, Huang S, Zhang A, Zhang Y (2021). Cilomilast ameliorates renal tubulointerstitial fibrosis by inhibiting the TGF-β1-Smad2/3 signaling pathway. Front Med.

[10] Wynn TA (2008). Cellular and molecular mechanisms of fibrosis. J Pathol.

[11] Wu Y, Tian Y-J, Le M-L, Zhang S-R, Zhang C, Huang M-X (2020). Discovery of novel selective and orally bioavailable phosphodiesterase-1 inhibitors for the efficient treatment of idiopathic pulmonary fibrosis. J Med Chem.

[12] Dubey A, Kumar N, Mishra A, Singh Y, Tiwari M (2020). Review on vinpocetine. Int J Pharm Life Sci.

[13] Nadeem RI, Ahmed HI, El-Sayeh BM (2018). Protective effect of vinpocetine against neurotoxicity of manganese in adult male rats. Naunyn Schmiedeberg Arch Pharmacol.

[14] Zhang C, Yan C (2020). Updates of recent vinpocetine research in treating cardiovascular diseases. J Cell Immunol.

[15] Poursalehi HR, Fekri MS, Far FS, Mandegari A, Izadi A, Mahmoodi R (2018). Early and late preventive effect of Nigella sativa on the bleomycin-induced pulmonary fibrosis in rats: An experimental study. Avicenna J Phytomed.

[16] Balaha M, Ahmed N, Geddawy A, Kandeel S (2021). Fraxetin prevented sodium fluoride-induced chronic pancreatitis in rats: Role of anti-inflammatory, antioxidant, antifibrotic and anti-apoptotic activities. Int Immunopharmacol.

[17] Balaha MF, Tanaka H, Yamashita H, Abdel Rahman MN, Inagaki N (2012). Oral Nigella sativa oil ameliorates ovalbumin-induced bronchial asthma in mice. Int Immunopharmacol.

[18] Pesce AJ (1987). Lactate dehydrogenase. Methods in Clinical Chemistry.

[19] Gornall AG, Bardawill CJ, David MM (1949). Determination of serum proteins by means of the biuret reaction. J Biol Chem.

[20] Ellman GL (1959). Tissue sulfhydryl groups. Arch Biochem Biophys.

[21] Nishikimi M, Rao NA, Yagi K (1972). The occurrence of superoxide anion in the reaction of reduced phenazine methosulfate and molecular oxygen. Biochem Biophys Res Commun.

[22] Ohkawa H, Ohishi N, Yagi K (1979). Assay for lipid peroxides in animal tissues by thiobarbituric acid reaction. Anal Biochem.

[23] Aebi H (1984). Catalase in vitro. Methods Enzymol.

[24] Reddy GK, Enwemeka CS (1996). A simplified method for the analysis of hydroxyproline in biological tissues. Clin Biochem.

[25] Suvarna KS, Layton C, Bancroft JD (2018). Bancroft’s theory and practice of histological techniques.

[26] Hübner R-H, Gitter W, Eddine El Mokhtari N, Mathiak M, Both M, Bolte H (2008). Standardized quantification of pulmonary fibrosis in histological samples. Biotechniques.

[27] Gagnon L, Leduc M, Grouix B, Tremblay M, Sarra-Bournet F, Laverdure A (2016). C74 models and methods in lung biology: Oral treatment with Pbi-4050 reduces plasminogen activator inhibitor-1 (pai-1), alpha-smooth muscle actin ([alpha]-sma) and collagen expression in human fibroblasts and mice fibrotic lungs. Am J Respir Crit Care Med.

[28] Matsuyama S, Karim MR, Izawa T, Kuwamura M, Yamate J (2018). Immunohistochemical analyses of the kinetics and distribution of macrophages in the developing rat kidney. J Toxicol Pathol.

[29] Pereira T, Naik S, Tamgadge A (2015). Quantitative evaluation of macrophage expression using CD68 in oral submucous fibrosis: an immunohistochemical study. Ann Medical Health Sci Res.

[30] Yanagihara T, Chong SG, Vierhout M, Hirota JA, Ask K, Kolb M (2020). Current models of pulmonary fibrosis for future drug discovery efforts. Expert Opin Drug Discov.

[31] Kolb P, Upagupta C, Vierhout M, Ayaub E, Bellaye PS, Gauldie J (2020). The importance of interventional timing in the bleomycin model of pulmonary fibrosis. Eur Respir J.

[32] Kaul S, Kaur I, Jakhar D, Edigin E, Caldito EG (2021). The diverse methods of bleomycin delivery in cutaneous warts: A literature review. Dermatol Ther.

[33] Jenkins RG, Moore BB, Chambers RC, Eickelberg O, Königshoff M, Kolb M (2017). An official American Thoracic Society workshop report: Use of animal models for the preclinical assessment of potential therapies for pulmonary fibrosis. Am J Respir Cell Mol Biol.

[34] Moore BB, Hogaboam CM (2008). Murine models of pulmonary fibrosis. A J Physiol Lung Cell Mol Physiol.

[35] Izbicki G, Segel M, Christensen T, Conner M, Breuer R (2002). Time course of bleomycin‐induced lung fibrosis. Int J Exp Pathol.

[36] Tavares LA, Rezende AA, Santos JL, Estevam CS, Silva AM, Schneider JK (2021). Cymbopogon winterianus essential oil attenuates bleomycin-induced pulmonary fibrosis in a murine model. Pharmaceutics.

[37] Dias HB, de Oliveira JR, Donadio MVF, Kimura S (2019). Fructose-1, 6-bisphosphate prevents pulmonary fibrosis by regulating extracellular matrix deposition and inducing phenotype reversal of lung myofibroblasts. PloS One.

[38] Nekrassov V, Sitges Ma (2000). Vinpocetine protects from aminoglycoside antibiotic-induced hearing loss in guinea pig in vivo. Brain Res.

[39] Kseibati MO, Sharawy MH, Salem HA (2020). Chrysin mitigates bleomycin-induced pulmonary fibrosis in rats through regulating inflammation, oxidative stress, and hypoxia. Int Immunopharmacol.

[40] Hosseini SA, Zahedipour F, Sathyapalan T, Jamialahmadi T, Sahebkar A (2021). Pulmonary fibrosis: Therapeutic and mechanistic insights into the role of phytochemicals. BioFactors.

[41] Huai B, Ding J (2020). Atractylenolide III attenuates bleomycin-induced experimental pulmonary fibrosis and oxidative stress in rat model via Nrf2/NQO1/HO-1 pathway activation. Immunopharmacol Immunotoxicol.

[42] Luhach K, Kulkarni GT, Singh VP, Sharma B (2021). Vinpocetine amended prenatal valproic acid induced features of ASD possibly by altering markers of neuronal function, inflammation, and oxidative stress. Autism Res.

[43] Ren L, Yang C, Dou Y, Zhan R, Sun Y, Yu Y (2017). MiR-541-5p regulates lung fibrosis by targeting cyclic nucleotide phosphodiesterase 1A. Exp Lung Res.

[44] Essam RM, Ahmed LA, Abdelsalam RM, El-Khatib AS (2019). Phosphodiestrase-1 and 4 inhibitors ameliorate liver fibrosis in rats: Modulation of cAMP/CREB/TLR4 inflammatory and fibrogenic pathways. Life Sci.

